# Lessons learned from a pilot program in the United Arab Emirates to improve resident physical examination skills

**DOI:** 10.5116/ijme.5e88.be99

**Published:** 2020-04-20

**Authors:** Halah Ibrahim, Thana Harhara, Reima Al Marshoodi, Ashraf Kamour, Satish C. Nair

**Affiliations:** 1 Department of Medicine, Sheikh Khalifa Medical City, Abu Dhabi, UAE; 2 Department of Academic Affairs, Tawam Hospital, Al Ain, UAE

**Keywords:** Pilot program, United Arab Emirates, resident physical examination skills

## To the Editor

A comprehensive and accurate history and physical examination is essential for clinical reasoning and forms the cornerstone of the doctor-patient relationship.  Incomplete or inaccurate history taking and physical examination skills may lead to excessive reliance on laboratory and imaging tests, delayed diagnoses, and potentially harmful consequences for patients.  Despite the importance, the teaching of physical exam techniques has become considerably less frequent over the past several decades.^1^  Clinical skills of medical trainees have been shown to directly correlate with the amount of time they spend with faculty examining patients.^2^  Yet, research shows that trainees rarely have the opportunity to see their faculty demonstrate an appropriate history and physical examinations.^3^  In a United States-based survey of medical students, nearly one third reported that their senior physicians rarely or never saw patients with them during daily ward rounds.^3^  This has resulted in a deterioration of clinical skills, with studies noting that faculty observations of trainee physical examinations reveal a high number of errors.^4^

As leaders of an international Internal Medicine physician training program in the United Arab Emirates, we must meet regulations of the Arab Board of Medical Specialties and the United States-based Accreditation Council for Graduate Medical Education- International (ACGME-I).  Arab Board graduation and licensure requirements mandate that our senior trainees pass a high stakes Objective Structured Clinical Examination (OSCE) that focuses primarily on physical exam skills.  Over the past five years, our program’s pass rates have been low.  Prior to the April 2019 OSCE, we developed a six-week physical examination review course as a pilot program.  In this paper, we share lessons learned from this experience with the hope of helping others avoid similar disappointing outcomes.

The pilot program consisted of late afternoon teaching sessions, lasting 45 to 60 minutes each, which were scheduled twice weekly.  Each session focused on a single organ system, and trainees practiced their clinical skills and detection of physical signs.  Patient volunteers were recruited from the inpatient medical unit based on underlying disease pathology and clinical findings.  In small groups of three to four, trainees performed a focused physical examination on an inpatient volunteer under the supervision of a senior clinician, who provided immediate feedback and demonstrated techniques as needed.  We were encouraged by studies demonstrating feasibility and success from similar educational programs.^5^  We felt strongly that teaching clinical skills should be patient-centered and led by faculty who can role model, assess, and provide real-time feedback, facilitating reflection-in-action.  A small group debrief at the end of each session further explored skill strengths and deficiencies, provided an opportunity for reflection-on-action, and helped trainees to develop personal learning goals.

We were surprised by difficulties faced both in the process and in the outcome of the review course. We had anticipated reluctance from our busy clinicians to take on additional teaching responsibility, albeit temporary, and did, in fact, sometimes find it difficult to ensure senior clinician involvement. When questioned about their hesitation, most faculty reported excessive clinical workload, while some complained about unfamiliarity with OSCE formats and the lack of checklists to guide the sessions.  Irrespective of faculty issues, trainee engagement was high and verbal feedback was positive. Since the sessions were optional, we were initially concerned that trainee participation in an after-hours activity would below.  However, we were pleased that trainee turn out was consistently high, and they were eager to perform and receive feedback on their clinical skills. During debriefing sessions, the trainees perceived great benefit from the extra teaching and felt that their clinical skills had improved significantly.  Yet, a survey of trainees on the morning of the licensing OSCE revealed that there were no significant differences in confidence levels or anxiety between trainees who participated in the pilot course and those who did not.  Further, much to our surprise, despite the extensive time and resource investment, pass rates remained low and were not significantly higher for the course participants.

The poor OSCE performance was disheartening for both the trainees and teaching faculty.  As program leaders, we were particularly discouraged by the outcome.  Several lessons were learned during this process.  First, we needed to acknowledge that clinical training in today’s busy academic medical centers does not naturally lend itself to teaching physical examination techniques.  Rapid throughput of patients through the hospital and excessive administrative burdens have significantly interfered with patient care time, with one study revealing that US hospitalists can spend as little as 18% of their shift with patients.^3^  Senior clinicians, who should serve as leaders of clinical skills training, face pressure to generate revenue through patient care activities and research, leaving less time for education.  As such, there must be a concerted effort to inculcate physical examinations into daily ward rounds indeed.  We also learned that our trainees highly value the opportunity to demonstrate and receive feedback on physical examination techniques.  They openly lament the erosion of bedside teaching and relish direct, hands-on educational activities that improve their clinical skills.  Perhaps the most important insight gained was that educational activities cannot exclusively revolve around the learners, but must consider faculty interest, availability, training and expertise. Several studies have shown that practicing physicians lack confidence in some of their physical exam skills and that even academic faculty have deficiencies in physical examination techniques.^6^^,^^7^  It is possible that some of the reluctance we faced was due to faculty discomfort with their own clinical exam skills.  In addition, not all faculty have teaching experience with the OSCE format, and the lack of preparation and checklists proved to be a barrier.  In planning a future faculty development program to build expertise around clinical skills, we are considering a two-phase program that begins with a traditional train-the-trainer model to build local faculty expertise.  These clinicians can then facilitate a co-learning model in which recent graduates and junior faculty and trainees are taught together.  This approach would require fewer content experts and avoid delays in the implementation of a longitudinal clinical skills curriculum.

In conclusion, the deterioration of physical examination skills is a serious concern.  Today’s faculty likely trained and practiced in an era where physical examinations were not emphasized and may lack confidence in their own clinical exam skills, contributing to a downward spiral.  Faculty development is essential so that daily ward rounds can again become an essential venue for clinician educators to role model and teach proper physical examination techniques during their everyday work.

### Conflict of Interest

The author declares that there are no conflicts of interest.
